# Efficacy of estradiol-dydrogesterone and auto-crosslinked hyaluronan gel in preventing intrauterine adhesions following missed miscarriage curettage: a retrospective observational study

**DOI:** 10.3389/frph.2025.1602451

**Published:** 2025-07-14

**Authors:** Huilin Sheng, Mengyun Sui, Lin Zhang, Jingfang Shi, Long Xue

**Affiliations:** ^1^ Department of Family Planning, Shanghai Putuo District Maternity and Infant Hospital, Shanghai, China; ^2^ Division of Chronic Non-Communicable Diseases and Injury Prevention, Shanghai Municipal Center for Disease Control and Prevention, Shanghai, China; ^3^ Department of Medical Affairs, Huashan Hospital, Fudan University, Shanghai, China

**Keywords:** missed miscarriage, intrauterine adhesions, estradiol-dydrogesterone, auto-crosslinked hyaluronan gel, curettage

## Abstract

**Objective:**

Curettage is a common treatment for missed abortion (MA). However, intrauterine adhesions (IUA) are a major postoperative problem that can lead to infertility and menstrual abnormalities. This study aimed to evaluate the effectiveness of estradiol-dydrogesterone (ED), auto-crosslinked hyaluronan (AH) gel, and their combination in preventing IUA following MA curettage.

**Methods:**

A retrospective cohort study was conducted between June 2022 and December 2023. A total of 284 women following MA curettage were included, with 265 completing follow-up assessments. Participants were divided into four groups: (1) ED group (oral estradiol-dydrogesterone therapy), (2) AH group (intrauterine AH gel application), (3) ED + AH group (combination therapy), and (4) control group (no intervention). IUA diagnosis was confirmed via hysteroscopy.

**Results:**

The incidence of IUA was significantly lower in the intervention groups compared to the control group (*p* = 0.001). The IUA rates were 7.94% in the ED group, 6.15% in the AH group, 5.71% in the ED + AH group, and 23.88% in the control group. Logistic regression analysis identified a significant reduction in IUA risk for patients receiving ED (OR = 0.17, *p* = 0.005), AH (OR = 0.13, *p* = 0.002), and ED + AH (OR = 0.15, *p* = 0.005) compared to the control group. Additionally, a history of three or more miscarriages was associated with a higher risk of IUA (OR = 4.34, *p* = 0.027).

**Conclusion:**

This study demonstrates that prophylactic treatment with ED and/or AH significantly reduces the incidence of IUA following curettage for MA. These findings underscore the importance of individualized endometrial repair and adhesion prevention strategies in preserving female reproductive health.

## 1 Introduction

Missed miscarriage (MA) is a common early pregnancy complication characterized by the in-utero demise of the embryo or fetus without expulsion of the gestational tissue (1). Due to the frequent absence of typical miscarriage symptoms—such as vaginal bleeding and cramping—diagnosis is often delayed (2). MA is estimated to occur in approximately 13.4%–20% of all pregnancies (3–5). The World Health Organization (WHO) recommends surgical intervention when there is incomplete evacuation of the uterine contents following a MA (2). Although surgical management is generally effective in mitigating complications associated with miscarriage, it carries inherent risks. Notably, intrauterine adhesion (IUA) formation represents a significant postoperative complication, with post-miscarriage curettage being a dominant risk factor (6, 7).A review of 1,856 IUA cases revealed that 66.7% occurred following curettage (8). IUA can lead to various reproductive health issues, including infertility, menstrual irregularities, and recurrent miscarriages (9). As such, the prevention of IUA following curettage for MA has garnered considerable global research attention, with multiple approaches being evaluated (10–15). However, no universally accepted standard treatment has emerged (16), and further research is warranted to identify the most effective preventive strategy (9). aims to evaluate the clinical efficacy of estradiol/dydrogesterone tablets (ED), auto-crosslinked hyaluronan gel (AH), and their combination in preventing IUA after MA curettage, with the ultimate goal of protecting female reproductive health.

## 2 Methods

### 2.1 Study design and setting

A retrospective observational cohort study was conducted at the Shanghai Putuo District Maternal and Child Health Centre in China. This retrospective study did not randomize participants; group allocation reflected actual clinical management. Data were collected from patients who underwent curettage for MA between June 2022 and December 2023. Follow-up assessments were performed at three months post-surgery and during the early postmenstrual phase (3–7 days after menstruation). The study was registered in the Chinese Clinical Trial Registry (Registration number: ChiCTR2300078906).

### 2.2 Participants

Between June 2022 and December 2023, 284 women meeting the diagnostic criteria for MA, as outlined in the Expert Consensus on the Treatment of MA (1), were included. Of these, 265 women were successfully followed up via telephone or outpatient visits, while 19 were excluded due to loss to follow-up.

### 2.3 Treatment protocol and preventive interventions

Preparatively, detailed medical histories were obtained and patients received standardized health education. Two hours before curettage, all patients were received 0.4 mg misoprostol (0.2 mg/tablet) for cervical priming (17). Under conventional intravenous anesthesia and ultrasonic monitoring, experienced senior doctors performed the curettage, and all specimens were submitted for pathological examination (1). Postoperatively, all patients received antibiotics to infection prophylaxis (18). Based on the preventive interventions for IUA, women were assigned to four groups:

(1) **ED group:** Women began oral ED therapy (combination of 2 mg estradiol and dydrogesterone 10 mg per tablet) on the night of the operation (one tablet per day for 28 days). Following one treatment course, therapy was resumed on the third day after menstrual recovery for three consecutive courses.
(2) **AH group:** Women received an intrauterine injection of 3.0 ml AH (3.0 ml/branch) immediately after operation.
(3) **ED** **+** **AH group**: Women received both the intrauterine injection of AH and oral ED therapy as described above.
(4) **Control group:** Women received no additional perioperative intervention.

### 2.4 Research variables

#### 2.4.1 Outcome variables

All diagnosed IUA presented with menstrual abnormalities (amenorrhea or hypomenorrhea), consistent with the Chinese Expert Consensus on the Clinical Diagnosis and Treatment of Intrauterine Adhesions (19). Women were followed up by hysteroscopy at three months after surgery or during the early postmenstrual phase (3–7 days after menses). For those presenting with abnormal menstruation or lack of menstrual recovery one-month post-operation, a vaginal ultrasound was performed. Hysteroscopy was then indicated if ultrasound findings revealed an endometrial thickness of less than 6 mm or suspected adhesions.

#### 2.4.2 Control variable

The study identified several covariates based on previous research and data availability, including socio-demographic factors (age, education, BMI) and reproductive characteristics (number of miscarriages and duration of amenorrhea). In this study, duration of amenorrhea was selected over gestational age due to its superior precision in quantifying endometrial exposure to retained tissue and to avoid potential multicollinearity arising from including both variables in the model.

### 2.5 Statistical analysis

Data analysis was performed using Stata 16.0 (StataCrop, USA). Continuous data with a normal distribution were presented as mean ± standard deviation (χ̅ ± s), and one-way analysis of variance was employed for comparisons among the four groups. Categorical data were expressed as absolute numbers and percentages, with the Fisher exact test applied for groups comparisons. A multivariable logistic regression model was used to evaluate the effects of different preventive interventions and other potential risk factors on the incidence of IUA following MA curettage, a multivariable logistic regression model was applied. A *p* < 0.05 was considered statistically significant.

### 2.6 Efforts to address bias

Data were extracted from the hospital's electronic medical records and follow-up documentation. To minimize selection bias, all eligible women during the study period were included. Hysteroscopic evaluations were performed by experienced clinicians who were blinded to the preventive interventions' allocation, and ultrasound assessments were standardized using the same equipment and protocols t across all women. Outcome assessors were also blinded to preventive intervention groups, and baseline demographic and reproductive characteristics were recorded to adjust for potential confounders during statistical analyses.

### 2.7 Ethical statement

The study was conducted in accordance with the principles of informed consent, voluntary participation, confidentiality, and non-maleficence. Informed consent was obtained from all participants (or their legal guardians), and patient data were anonymized using codes. The study protocol was approved by the Ethics Committee of the Shanghai Putuo District Maternal and Child Health Centre (approval no.: PFYLL-2021003).

## 3 Result

### 3.1 General characteristics

A total of 284 women met the inclusion criteria between June 2022 and December 2023. During the follow-up period, 19 women (6.69%) were lost due to travel, relocation, failure to respond to telephone calls, or lack of cooperation. Losses were distributed as follows: 4 in the control group, 8 in the ED group, 6 in the AH group, and 1 in the ED + AH group. Ultimately, 265 women (93.31%) completed the follow-up, including 67 in the control group, 63 in the ED group, 65 in the AH group, and 70 in the ED + AH group. There were no significant differences among the groups regarding age, BMI, educational level, number of miscarriages, or duration of amenorrhea (all *p* > 0.05), as shown in Table 1.

**Table 1:** Comparison of the general features among the ED, AH, ED + AH and control group.

	Control Group (*n* = 67)	ED Group (*n* = 63)	AH Group (*n* = 65)	ED + AH Group (*n* = 70)	*χ*²/F	*p*
Age (`x ± s, years)	36.57 ± 6.09	35.51 ± 5.56	33.79 ± 9.56	35.67 ± 5.17	1.95	0.123
Educational level [%(*n*)]						
Less than lower secondary	29.23 (19)	25.40 (16)	32.86 (23)	34.33 (23)	4.54	0.604
Upper secondary/vocational	24.62 (16)	30.16 (19)	25.71 (18)	34.33 (23)		
Tertiary	46.15 (30)	44.44 (28)	41.43 (29)	31.34 (21)		
BMI (`x ± s, kg/m²)	21.46 ± 1.70	21.28 ± 2.23	21.01 ± 1.31	21.81 ± 1.88	2.32	0.076
Number of Miscarriages [%(*n*), times]						
0	26.15 (17)	14.29 (9)	31.43 (22)	26.87 (18)	9.38	0.403
1	36.92 (24)	44.44 (28)	40.00 (28)	47.76 (32)		
2	20.00 (13)	25.40 (16)	15.71 (11)	11.94 (8)		
≥3	16.92 (11)	15.87 (10)	12.86 (9)	13.43 (9)		
Duration of Amenorrhea (`x ± s, days)	66.37 ± 5.92	66.81 ± 5.38	65.83 ± 2.66	66.42 ± 5.74	0.43	0.730

BMI, body mass index; IUA, intrauterine adhesions; AH, auto-crosslinked hyaluronan gel; ED, estradiol/dydrogesterone tablets; ED + AH, estradiol/dydrogesterone tablets + auto-crosslinked hyaluronan gel.

### 3.2 Incidence of IUA at 3 months postoperatively

At the three-month follow-up, the incidence of IUA was compared among the four groups. The ED + AH group exhibited the lowest IUA incidence (4 cases, 5.71%), followed by the AH group (4 cases, 6.15%) and the ED group (5 cases, 7.94%). The control group demonstrated the highest incidence (16 cases, 23.88%). The differences in IUA incidence among the groups were statistically significant (*χ*^2^ = 15.58, *p* = 0.001).

### 3.3 Multivariable logistic regression analysis

A multivariable logistic regression analysis was performed with demographic characteristics (age, BMI, education) and reproductive characteristics (number of miscarriages, duration of amenorrhea) as explanatory variables, and the preventive method as the independent variable (coded as 0 = control, 1 = AH, 2 = ED, 3 = ED + AH). The analysis indicated that age, BMI, education, and duration of amenorrhea were not significantly associated with IUA. However, the number of miscarriages was positively correlated with IUA risk; notably, women with three or more miscarriages had a significantly higher incidence of IUA (OR = 4.34, *p* = 0.027). Preventive interventions significantly reduced IUA incidence compared to the control group, with OR values of 0.13, 0.17, and 0.15 for the AH, ED, and ED + AH groups, respectively (*p* = 0.002, 0.005, and 0.005), as shown in Table 2.

**Table 2:** Multivariable logistic regression analysis of IUA incidence.

Variable	OR	SE	Z	*p*	95% CI	
Age	1.01	0.04	0.37	0.713	0.94	1.09
BMI	1.06	0.13	0.45	0.653	0.83	1.35
Education						
Less than lower secondary	Ref.					
Upper secondary/vocational	0.31	0.21	−1.76	0.079	0.09	1.14
Tertiary	1.15	0.58	0.28	0.779	0.43	3.09
Number of miscarriages						
0	Ref.					
1	0.60	0.39	−0.78	0.433	0.17	2.16
2	2.13	1.48	1.09	0.275	0.55	8.28
≥3	4.34	2.87	2.22	0.027	1.19	15.87
Duration of Amenorrhea	1.07	0.05	1.67	0.094	0.99	1.17
Preventive IUA method						
Control Group	Ref.					
AH Group	0.13	0.08	−3.13	0.002	0.03	0.46
ED Group	0.17	0.11	−2.79	0.005	0.05	0.59
ED + AH Group	0.15	0.10	−2.83	0.005	0.04	0.56

BMI, body mass index; IUA, intrauterine adhesions; AH, auto-crosslinked hyaluronan gel; ED, estradiol/dydrogesterone tablets; ED + AH, estradiol/dydrogesterone tablets + auto-crosslinked hyaluronan gel; OR, odds ratio; SE, standard error; CI, confidence interval.

## 4 Discussion

Curettage performed for MA can result in endometrial damage and subsequent IUA, which may adversely affect fertility (7). This study demonstrated that the application of AH and/or ED significantly reduced the incidence of IUA following MA curettage. Specifically, the IUA incidence was 7.94%, 5.71%, 6.15%, and 23.88% in the ED group, ED + AH group, AH group, and control group, respectively, which consistent with the previously reported range of 3%–38% (11, 12, 20, 21). Multivariable logistic regression analysis indicated that an higher number of miscarriages, particularly three or more miscarriages, was significantly associated with increased IUA risk. In contrast, the use of prophylactic interventions (AH, ED, or their combination) markedly decreased this risk.

The beneficial effects of ED may be attributed to the pharmacological actions of its components. Estradiol promotes endometrial proliferation and repair following curettage, reducing fibrosis and enhancing angiogenesis, which supports the regeneration of stromal tissue, endometrial glands, and blood vessels. Dydrogesterone facilitates the transition of the endometrium to the secretory phase, accelerates menstrual recovery, and increases the viscosity of cervical mucus, thereby reducing infection risk and contributing to IUA prevention (12). Similarly, auto-crosslinked hyaluronan gel functions as a mechanical barrier that prevents adhesion formation between opposing endometrial surfaces. The gel is fully degraded and absorbed within 7–14 days, a timeframe that is critical for proper endometrial healing (14, 22). Previous studies have also reported that both ED and AH can modulate inflammatory responses, promote angiogenesis, and inhibit scar formation, thereby enhancing postoperative healing and preventing IUA (11, 12, 23, 24).

The observation that an increased number of miscarriages elevates the risk of IUA is consistent with prior research (25, 26). Mechanical trauma to the endometrium during curettage is a known precipitating factor for IUA (27, 28). In the context of pregnancy, the endometrial basal layer is highly vascularized and relatively fragile; repeated or deep curettage can damage this layer. Furthermore, the abrupt decline in sex hormones following pregnancy termination reduces angiogenesis and oxygen supply, thereby increasing adhesion factor secretion and delaying endometrial repair (27, 29). In this study, factors such as age, BMI, and menstrual irregularities were not significantly associated with IUA incidence, which corroborates previous findings (25).

Several limitations must be acknowledged. First, the observational study design precludes the establishment of causality between the preventive interventions and the reduced incidence of IUA. Although a significant association was observed, unmeasured factors—such as detailed reproductive and menstrual histories or the timing of topical protective measures—may have contributed to the outcomes. Second, Although patients with preexisting IUA were excluded, detailed classification of prior miscarriage management (e.g., spontaneous miscarriage without intervention vs. induced miscarriage with curettage) was not performed, potentially affecting baseline risk assessment. Third, the single-institution setting may limit the generalizability of the findings. Future multicenter studies are recommended to validate these results and further explore additional risk factors.

## 5 Conclusion

This study demonstrates that prophylactic treatment with ED and/or AH significantly reduces the incidence of IUA following curettage for MA. These findings underscore the importance of individualized endometrial repair and adhesion prevention strategies in preserving female reproductive health. Further clinical investigations are warranted to evaluate subsequent pregnancy outcomes and to refine prophylactic protocols.

## Data Availability

The raw data supporting the conclusions of this paper are provided by Shanghai Putuo District Maternity and Infant Hospital, Shanghai, China, further inquiries need to apply to hospital for approval. Requests to access the datasets should be directed to LX, xuelong@huashan.org.cn.
